# Work Reintegration After Chemotherapy in Patients With Early-Stage Breast Cancer: The RESTART Cross-Sectional Survey

**DOI:** 10.7759/cureus.99684

**Published:** 2025-12-20

**Authors:** Sandra Silva, Beatriz Belo, Adriana Soares, Cristiana Marques, Joana Marinho, Andreia Capela, Rita Canário, Cecília Carvalho, Viviana Teixeira, Raquel Monteiro

**Affiliations:** 1 Oncology, Unidade Local de Saúde Gaia/Espinho, Vila Nova de Gaia, PRT; 2 Psychology, Vocational Rehabilitation Centre, Vila Nova de Gaia, PRT; 3 Oncology, Unidade Local de Saúde Gaia / Espinho, Vila Nova de Gaia, PRT

**Keywords:** breast cancer, occupational health, quality of life, return to work, survivorship

## Abstract

Background: Return to work (RTW) is a key milestone for breast cancer survivors, reflecting financial stability, personal recovery, and social reintegration. However, this transition is often hindered by persistent physical, cognitive, and emotional sequelae of treatment. In this single-center exploratory study within the RESTART project, we aimed to evaluate return-to-work outcomes and identify clinical, psychosocial, and occupational factors associated with sick leave and prolonged work absence in women treated with chemotherapy for early-stage breast cancer.

Methods: We conducted a cross-sectional study in Portugal that included professionally active women (≤60 years) at least 12 months after completing chemotherapy. Data were collected from medical records and patient questionnaires addressing sociodemographic, clinical, and employment-related factors. Quality of life was assessed using the EORTC QLQ-BR23. Multivariable logistic regression was used to identify factors associated with sick leave and prolonged leave (>12 months).

Results: Seventy-one women were included (median age 52 years, interquartile range (IQR) 49-57). Most participants (59, 83.1%) took sick leave, usually starting at diagnosis, and 42 (71.2%) remained on leave after chemotherapy. At evaluation, 48 (81.4%) had returned to work, with a median time of 18 months from diagnosis (IQR 11-30) and 13 months from the end of chemotherapy (IQR 7-27). However, 7 (14.6%) transitioned to part-time work, 13 (27.1%) changed roles, and 11 (22.9%) reported income loss. The most common reasons for continued leave were fatigue (33, 82.5%), cognitive impairment (26, 72.2%), mood changes (20, 55.6%), and insufficient workplace adaptation (17, 53.1%). Physical exercise (34, 64.2%), psychological support (25, 47.2%), and physical therapy (25, 47.2%) were frequently reported as facilitators of return. In multivariable analysis, lower educational level (odds ratio (OR) 4.67, 95% confidence interval (CI) 1.07-20.5, *P *= 0.040) and higher physical job demands (OR 1.43, 95% CI 1.09-1.87, *P *= 0.009) were associated with persistent leave, while anti-human epidermal growth factor receptor 2 (anti-HER2) therapy showed a borderline association (OR 3.45, *P *= 0.074). Women on leave reported significantly worse quality of life, particularly in body image and breast and arm symptoms.

Conclusions: RTW after breast cancer is influenced by medical, occupational, and psychosocial factors. In this exploratory single-center sample, Portuguese survivors experienced substantial work disruption, underscoring the need for integrated survivorship care with vocational support. Interventions should focus on symptom management, employer engagement, and structured work capacity assessment. Public health strategies should acknowledge work reintegration as a core component of post-cancer recovery.

## Introduction

Over the past decades, the number of cancer survivors has steadily increased, in parallel with advances in early diagnosis and treatment [1]. As cancer increasingly becomes a chronic condition, it poses not only scientific and clinical challenges but also significant psychosocial implications for patients.

Employment is a crucial aspect of cancer survivorship, contributing not only to financial stability but also to psychological well-being, social identity, and overall independence [2]. However, many cancer survivors face significant challenges when attempting to return to work (RTW) or maintain their professional roles. Compared to the general population, they are approximately 40% more likely to be unemployed [3], which has led the Multinational Association of Supportive Care in Cancer (MASCC) to advocate for integrating RTW support into oncologic care [4].

Breast cancer is the most prevalent malignancy among women worldwide, and its survival rate is among the highest for early-stage cancers [5]. The median age at breast cancer diagnosis is 62 years [5], which is below the current retirement age in Portugal. As such, a substantial proportion of breast cancer survivors belong to the working-age population. Given this demographic profile and high survival rates, understanding RTW patterns is essential.

International evidence shows that RTW after breast cancer is highly variable. A 2014 systematic review including 26 studies reported one-year RTW rates ranging from 43% to 93%, with major barriers including low educational attainment, chemotherapy, physical limitations, and psychological distress [6]. More recent studies confirmed that breast cancer survivors often report reduced work ability compared to women without cancer [7-9] and that multidisciplinary rehabilitation strategies, combining physical, psychological, and vocational support, can significantly improve RTW outcomes [10].

While international studies have extensively explored work outcomes after cancer, national data remain limited. In Portugal, according to the Organization for Economic Co-operation and Development (OECD), survival rates for the most common cancers are above the European average [11]. A prospective cohort study published in 2019 found that, among 242 women employed before diagnosis, 26 became unemployed after treatment, 27 opted for early retirement, and 13 were on sick leave at the five-year follow-up [12]. Furthermore, a nationwide survey conducted in 2021 by the Portuguese League Against Cancer reported that breast cancer survivors often experienced delays in life projects (56%) and professional changes (20%), and highlighted the significant indirect economic impact of the disease, with lost wages estimated at more than €1.3 billion [13].

Given the substantial economic and personal consequences of employment disruption, it is essential to characterize RTW patterns among breast cancer survivors. Therefore, the primary aim of this study was to assess the rate and timing of RTW after chemotherapy for early-stage breast cancer. Secondary aims were to examine factors associated with prolonged sick leave and to explore clinical, psychosocial, and occupational contributors to work reintegration challenges.

This study was previously presented as an e-poster at the European Society for Medical Oncology (ESMO) Congress 2025, using a preliminary version of the dataset; the present manuscript includes the expanded and updated analysis.

## Materials and methods

Study design and setting

This was a single-center, cross-sectional observational study conducted at the Unidade Local de Saúde Gaia/Espinho in Porto, Portugal, between January 2024 and May 2025.

Study objectives

The primary outcome was the return-to-work rate and the median time to professional reintegration after diagnosis and after chemotherapy. Secondary outcomes included median duration of sick leave, employment changes (for example, job change, role modifications, working hours, or income), self-reported barriers and facilitators to RTW, quality of life, and predictors of sick leave and prolonged work absence.

Participants and eligibility criteria

The study population consisted of female breast cancer survivors who met the following inclusion criteria: aged ≤ 60 years at the time of inclusion; diagnosed with stage I to III breast cancer; treated with neoadjuvant and/or adjuvant chemotherapy; completion of chemotherapy at least 12 months before inclusion; professionally active at the time of cancer diagnosis; and able to understand the study procedures and provide informed consent. Participants were excluded if they had a suspected or confirmed recurrence of breast cancer or had a previous diagnosis of another malignancy, excluding breast cancer. A total of 75 eligible women who met the inclusion criteria were contacted during the recruitment period, of whom 71 agreed to participate, resulting in a response rate of 94.7%. Participation flow, therefore, reflected all patients who were reachable and willing to undergo assessment at least 12 months after completing chemotherapy.

Sample size calculation

A minimum sample size of 384 participants was estimated using a single-proportion formula, assuming a 50% prevalence for maximum variability with a 5% margin of error and a 95% confidence level. This calculation was based on the initial multicentric design of the RESTART project. However, the present manuscript reports only data from a single center, corresponding to a smaller sample that should be considered exploratory.

Data collection and instruments

An investigator collected clinical and pathological data through a review of medical records. Clinical staging was performed according to the eighth edition of the AJCC TNM classification system. Demographic information and employment-related data were collected using a questionnaire completed by breast cancer survivors. The instrument was designed specifically for this study by the research team, which included a neuropsychologist and a research and development assistant from the Gaia Professional Rehabilitation Center. 

Although not a formally validated occupational measure, the job demand component was intentionally developed to pragmatically capture the physical, cognitive, and emotional requirements of participants’ professional activities within the Portuguese labor context. To enhance consistency, all participants received standardized instructions and examples when completing the scale.

Occupational demands were assessed through three self-reported items in which participants rated the physical, cognitive, or mental, and emotional or psychological requirements of their professional activities on a numeric scale from one (“not demanding at all”) to ten (“extremely demanding”). Quality of life was assessed using the EORTC QLQ-BR23 [14], a breast cancer-specific quality-of-life questionnaire developed by the European Organization for Research and Treatment of Cancer.

Statistical analysis

Statistical analysis was performed using IBM SPSS Statistics, version 29. Descriptive statistics were used to summarize sociodemographic and clinical characteristics. Categorical variables were reported as frequencies and percentages, while continuous variables were presented as medians and interquartile ranges (IQR). Missing data were minimal and were handled using complete-case analysis, with no imputation performed. Logistic regression models were used to identify predictors of two binary outcomes: sick leave following the completion of active treatment, and prolonged sick leave lasting more than 12 months. Variables with p-values < 0.10 in univariable analysis were considered for inclusion in the multivariable models. Multicollinearity was checked, and no concerning correlations were identified among predictors. Model fit was assessed using standard goodness-of-fit statistics available for logistic regression. A p-value < 0.05 was considered statistically significant. Quality-of-life scores from the EORTC QLQ-BR23 questionnaire [14] were calculated and transformed to a 0-100 scale according to the EORTC Scoring Manual. Comparisons between groups were conducted using the Mann-Whitney U test.

Ethical considerations

The study protocol was approved by the Ethics Committee of the Unidade Local de Saúde Gaia/Espinho. All participants provided written informed consent before participation and were free to withdraw at any time without consequence. The study adhered to the principles of the Declaration of Helsinki and the European Medicines Agency guideline for good clinical practice. All data were anonymized before analysis, and identifiable information was accessible only to local investigators.

## Results

Participant characteristics at the time of diagnosis of breast cancer

A total of 71 women were included in the study, with a median age of 52 years (IQR: 49-57). The majority resided in the district of Porto (*n* = 57, 80.3%), particularly in Vila Nova de Gaia (*n* = 52, 73.2%). Most participants were either married or in a common-law partnership (*n* = 56; 78.9%) and reported having at least one dependent (*n* = 49; 69.0%). Regarding educational level, 21 (29.6%) had completed secondary education, 20 (28.2%) held a university degree, and another 20 (28.2%) had completed grades 5 to 9. The most frequently reported occupations were administrative assistant (*n* = 8, 11.3%), domestic worker (*n* = 8, 11.3%), seamstress (*n* = 5, 7.0%), teacher (*n* = 4, 5.6%), nurse (*n* = 4, 5.6%), kitchen assistant (*n* = 3, 4.2%), and factory worker (*n* = 3, 4.2%). The median physical demand of participants’ jobs was 7 (IQR: 5-9), while the median cognitive demand was 8 (IQR: 6-9), and the median emotional or psychological demand was 9 (IQR: 6-9), based on self-reported scores on a 0-10 scale (0 = none, 10 = very high). Most participants were employed in the private sector (*n* = 53, 74.6%), worked full-time (*n* = 61, 85.9%), and held a permanent contract (*n*= 48, 67.6%). The most frequently reported gross monthly salary corresponded to the national minimum wage (*n* = 30, 42.3%), and household income most commonly ranged between €1,000 and €2,000 (*n* = 17, 23.9%). Additional sociodemographic and occupational characteristics are shown in Table 1.

**Table 1:** Baseline sociodemographic and occupational characteristics of the study population (n = 71). Minor dependents: individuals <18 years; adult dependents: individuals ≥18 years requiring care. Job demands were self-rated on a 0-10 scale (0 = none, 10 = very high). Percentages may not sum to 100% due to rounding or missing data. n, number; %, percentage; IQR, interquartile range

Category	
Numerical variables	
Age, median years (IQR)	52 (49-57)
Sociodemographic	
Marital status, *n* (%)	
Married/Partnered	56 (78.9)
Single/Divorced/Widowed	15 (21.1)
Dependent care responsibilities, *n* (%)	49 (69.0)
Adults	29 (58.0)
Minors	17 (34.0)
Both	4 (8.0)
District of residence, *n* (%)	
Porto	57 (80.3)
Aveiro	13 (18.3)
Vila Real	1 (1.4)
Education Level, *n* (%)	
≤4th grade	10 (14.1)
5th-9th grade	20 (28.2)
10th-12th grade	21 (29.6)
University degree	20 (28.2)
Occupational	
Main occupation, *n* (%)	
Administrative assistant	8 (11.3)
Domestic worker	8 (11.3)
Seamstress	5 (7.0)
Teacher	4 (5.6)
Nurse	4 (5.6)
Others	42 (59.2)
Employment sector, *n* (%)	
Private	53 (74.6)
Public	18 (25.4)
Contract type, *n* (%)	
Permanent contract	48 (67.6)
Temporary contract	11 (15.5)
Self-employed worker	6 (8.5)
Worker without social benefits	4 (5.6)
Freelancer	2 (2.8)
Working hours, *n* (%)	
Full-time	61 (85.9)
Part-time	10 (14.1)
Gross monthly salary (€), *n* (%)	
National minimum wage	2 (2.8)
National minimum wage	30 (42.3)
<1,000	17 (23.9)
1,000-2,000	17 (23.9)
>2,000	4 (5.6)

Clinical characteristics

Most participants were diagnosed with stage II breast cancer (*n* = 39, 54.9%). The most frequent tumor subtype was Luminal HER2-negative (*n* = 40, 56.3%), followed by Luminal HER2-positive (*n* = 17, 23.9%). Mastectomy was performed in 44 (62%) patients, whereas 27 (38.0%) underwent breast-conserving surgery. Sentinel lymph node biopsy was carried out in 47 (66.2%) patients, while 24 (33.8%) required axillary lymph node dissection.

Neoadjuvant chemotherapy was administered to 41 (57.7%) women, and 30 (42.3%) received adjuvant chemotherapy. Anti-HER2 therapy was given to 25 (35.2%) patients. Among those receiving adjuvant endocrine therapy (*n* = 57, 80.3%), 34 (59.6%) were treated with tamoxifen and 23 (40.3%) with aromatase inhibitors. Ovarian suppression or oophorectomy was performed in 23 (37.1%) of participants, and adjuvant radiotherapy was delivered to 49 (69.0%) patients. Additional clinical characteristics are presented in Table 2.

**Table 2:** Baseline clinical characteristics (n = 71). Cancer stage is classified according to the AJCC 8th edition. Percentages may not total 100% due to rounding. HR, hormone receptor; HER2, human epidermal growth factor receptor 2

Category, *n* (%)	
Cancer stage	
I	15 (21.2)
II	39 (54.9)
III	17 (23.9)
Tumor subtype	
HR+ HER2-	40 (56.3)
HR+ HER2+	17 (23.9)
HR- HER2+	8 (11.3)
Triple negative	5 (7.0)
Breast surgery	
Mastectomy	44 (62.0)
Breast-conserving surgery	27 (38.0)
Axillary surgery	
Sentinel lymph node biopsy	47 (66.2)
Axillary lymph node dissection	24 (33.8)
Chemotherapy setting	
Neoadjuvant	41 (57.7)
Adjuvant	30 (42.3)
Chemotherapy regimen	
Taxane and anthracycline	52 (73.2)
Taxane only	10 (14.1)
Platinum, taxane, and anthracycline	8 (11.3)
Taxane and platinum	1 (1.4)
Adjuvant radiotherapy	49 (69.0)
Adjuvant endocrine therapy	57 (80.3)
Tamoxifen	34 (59.6)
Aromatase inhibitor	23 (40.4)
Ovarian suppression or oophorectomy	23 (37.1)
Anti-HER2 therapy	25 (35.2)

RTW outcomes

After a median follow-up of 40 months (IQR: 30-65), 59 (83.1%) participants went on sick leave at some point following their diagnosis. Among these, 50 (84.7%) initiated sick leave at the time of diagnosis, and 42 (71.2%) remained on leave after completing chemotherapy, during surveillance or adjuvant endocrine therapy. In contrast, 9 (15.3%) began sick leave only after completing chemotherapy. Of those who took leave, 48 (81.4%) had returned to work by the time of data collection, whereas 11 (18.6%) had not resumed employment. The median time from diagnosis to RTW was 18 months (IQR: 11-30) and 13 months (IQR: 7-27) after completing chemotherapy. Following work reintegration, 7 (14.6%) transitioned from full-time to part-time work, 13 (27.1%) changed their job role or position, 11 (22.9%) reported reduced income, and 7 (14.6%) moved to a different employer. Additional return-to-work outcomes are presented in Table 3.

**Table 3:** Return-to-work outcomes among participants who took sick leave (n = 59). Percentages refer to participants who took sick leave (n = 59) and may not total 100% due to rounding. Note: This table includes only participants who returned to work (n = 59), as these outcomes are specific to this subgroup. RTW, return to work; ChT, chemotherapy; IQR, interquartile range

Outcome	*n* = 59
Timing of sick leave, *n* (%)	
Started at diagnosis	50 (84.7)
Persisted after active treatment	42 (71.2)
Only post-ChT	9 (15.3)
RTW, *n* (%)	
Yes	48 (81.4)
No	11 (18.6)
Time from diagnosis to RTW (months), median (IQR)	18 (11–30)
Time from end of ChT to RTW (months), median (IQR)	13 (7–27)
Transitioned to part-time work, *n* (%)	7 (14.6)
Changed job role or position, *n* (%)	13 (27.1)
Reported income decrease, *n* (%)	11 (22.9)
Job/employer change, *n* (%)	7 (14.6)

**Figure 1:**
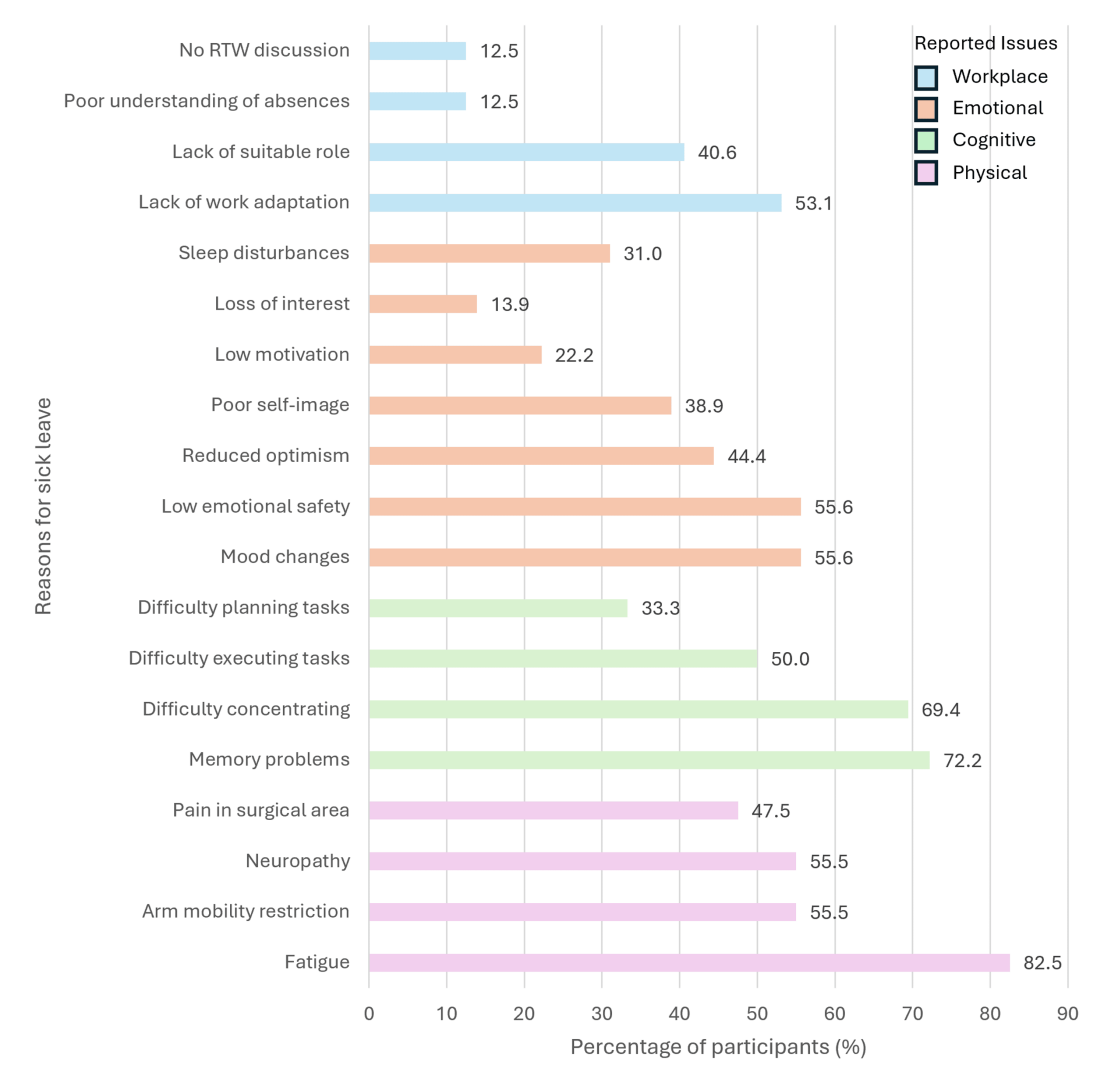
Self-reported reasons for initiation or maintenance of sick leave among breast cancer survivors (n = 59). Participants identified the symptoms or challenges that contributed to their initiation or maintenance of sick leave. Reported issues were categorized into four domains: physical, cognitive, emotional, and workplace-related. Multiple responses were allowed. Note: This figure includes only participants who were on sick leave (*n* = 59), as these reasons are specific to this subgroup. RTW, return to work

Regarding the reasons for taking sick leave, 40 (67.8%) reported physical symptoms, most frequently fatigue (*n* = 33, 82.5%), pain in the surgical area (*n* = 19, 47.5%), restricted arm mobility (*n* = 22, 55.0%), neuropathy (*n* = 22, 55.0%), and, less commonly, lymphedema (*n* = 3, 7.7%). Cognitive complaints were reported by 36 (61%), including memory impairment (*n* = 26, 72.2%), difficulties with attention and concentration (*n* =25, 69.4%), difficulty executing planned tasks (*n* =18, 50.0%) and challenges in planning activities (*n* =12, 33.3%). Emotional symptoms were reported by 36 (61.0%); these included recurrent mood changes (*n* = 20, 55.6%), decreased sense of emotional safety (*n* = 20, 55.6%), reduced optimism (*n* =16, 44.4%), negative self-image (*n* =14, 38.9%), sleep disturbances (*n* = 22, 31%), decreased initiative or motivation (*n* = 8, 22.2%) and loss of interest in previously enjoyable activities (*n* = 5, 13.9%). Employment-related factors were identified by 32 (54.0%), including lack of adaptation of work conditions due to functional limitations (*n* = 17, 53.1%), absence of suitable job functions (*n* = 13, 40.6%), limited understanding regarding absences for medical appointments (*n* = 4, 12.5%), and lack of discussion about the possibility of returning to work (*n* = 4, 12.5%). These findings are shown in Figure 1.

**Figure 2:**
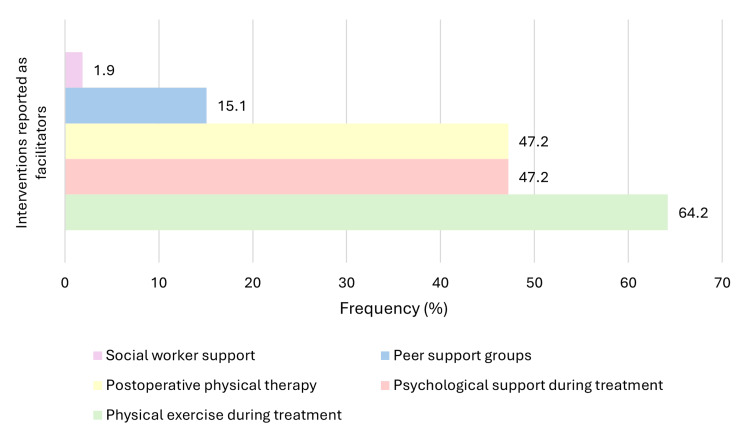
Self-reported facilitators of return to work among breast cancer survivors (n = 48). Participants identified the factors they perceived as supporting their return to work after treatment, including physical exercise, psychological support, and physical therapy. Multiple responses were allowed. Note: This figure includes only participants who both took sick leave and subsequently returned to work (*n* = 48), as these outcomes are specific to this subgroup.

**Figure 3:**
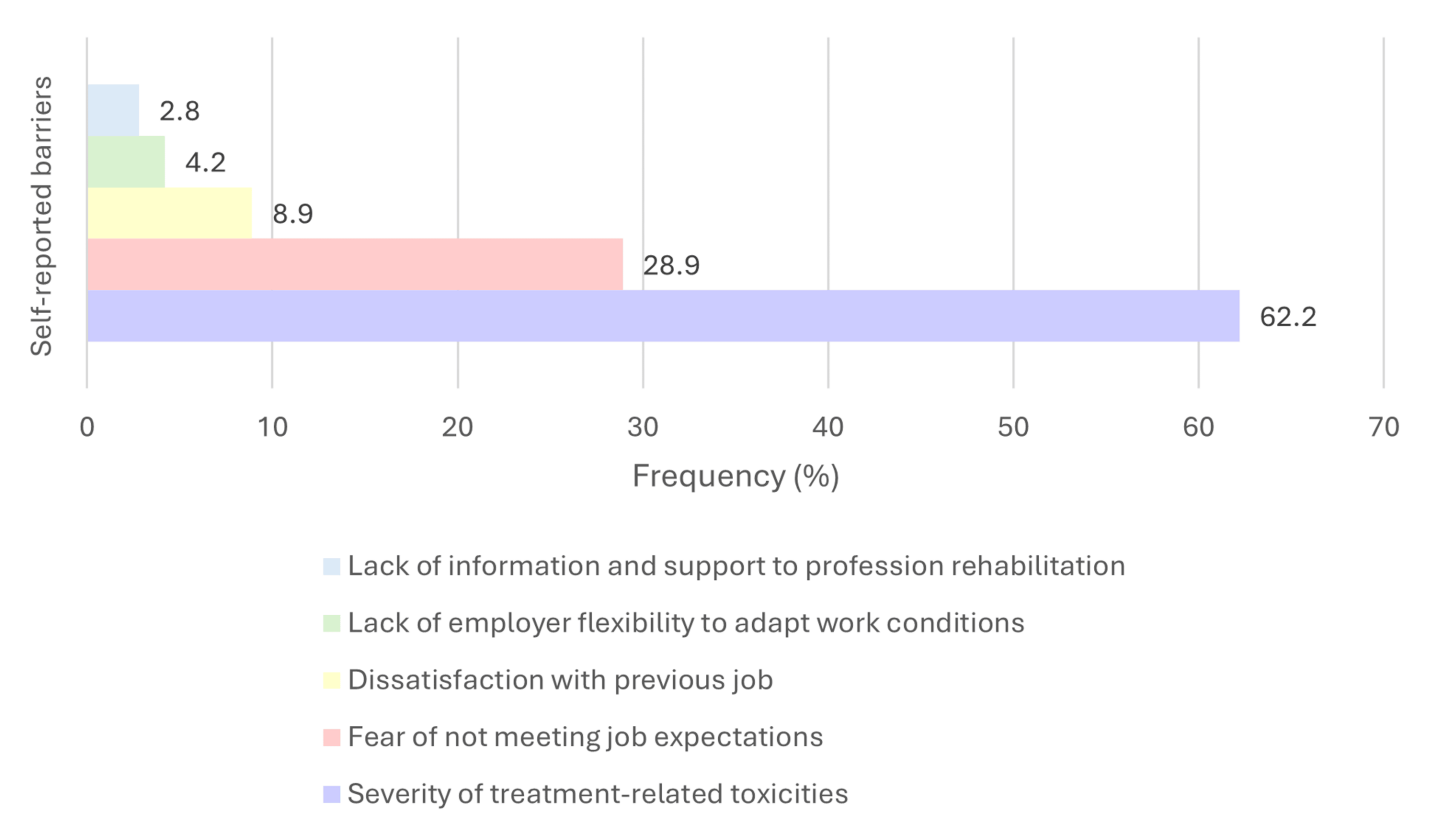
Self-reported barriers to return to work among breast cancer survivors (n = 59). Participants identified perceived obstacles that hindered their ability to return to work after treatment. Multiple responses were allowed. Note: This figure includes only participants who took sick leave (*n* = 59), as barriers to return to work were assessed only within this subgroup.

**Figure 4:**
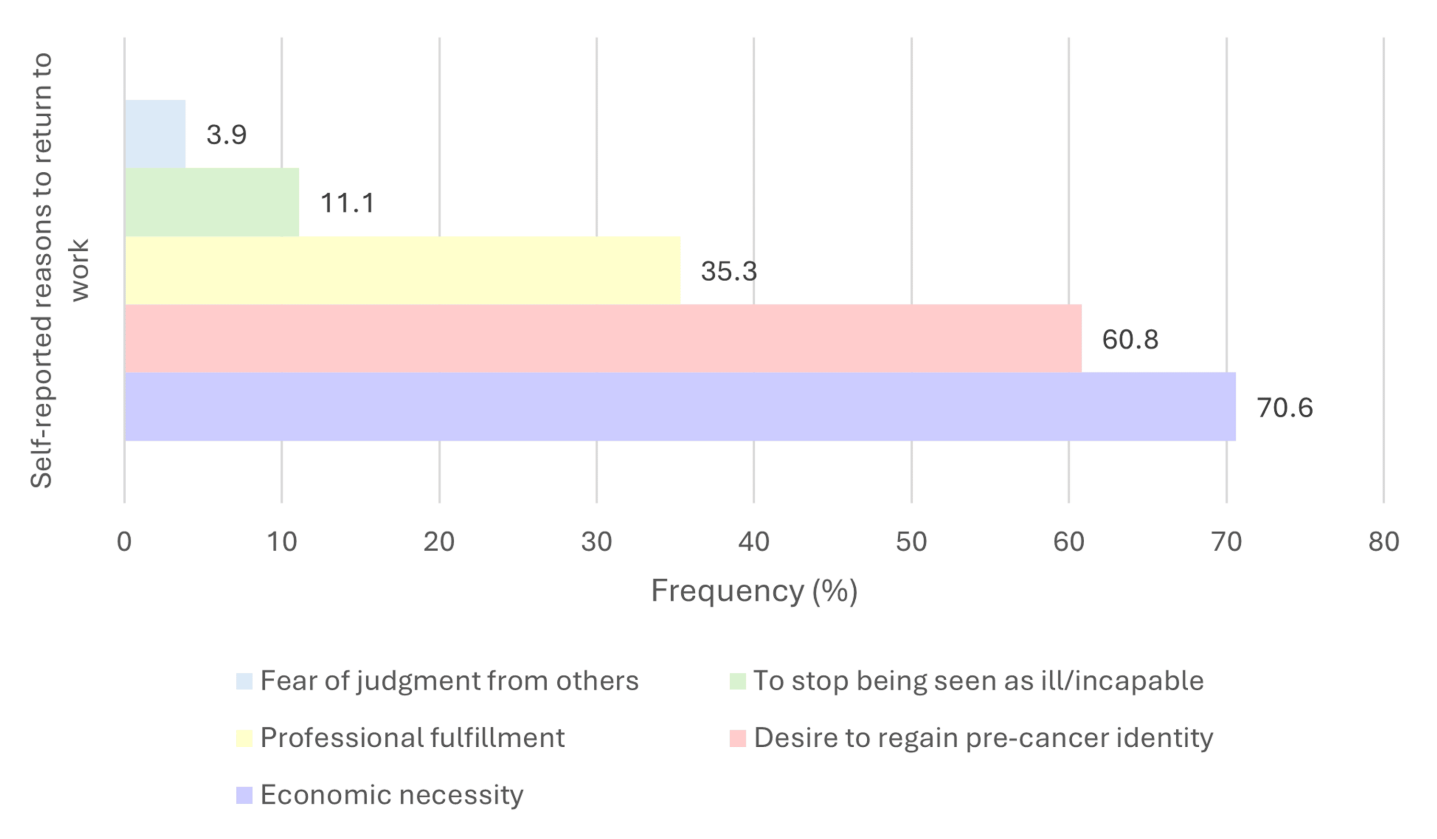
Self-reported main reasons for returning to work among breast cancer survivors (n = 48). Participants selected their primary motivations for resuming work after cancer treatment. Multiple responses were allowed. Note: This figure includes only participants who returned to work (*n* = 48), as motivations for returning were assessed exclusively within this subgroup.

Participants also reported several factors that facilitated RTW, most commonly physical exercise during treatment (*n* = 34, 64.2%), psychological support (*n* = 25, 47.2%), and postoperative physical therapy (*n* = 25, 47.2%). These facilitators are displayed in Figure 2. The main barriers to returning to work included treatment-related toxicities (*n* = 28, 62.2%) and fear of not meeting job expectations (*n* = 13, 28.9%), as illustrated in Figure 3. The most frequently cited motivations for returning to work were economic necessity (*n* = 36, 70.6%), the desire to reconnect with their pre-cancer identity (*n* = 31, 60.8%), and professional fulfillment (*n* = 18, 35.3%), shown in Figure 4.

Univariable and multivariable analysis of sick leave predictors

Univariable analyses identified several factors associated with sick leave after treatment, including lower educational level (OR 2.89, 95% CI 1.05-7.94, *P* = 0.041), permanent employment contract (OR 0.29, 95% CI 0.10-0.82, *P* = 0.020), and higher physical (OR 1.56, 95% CI 1.23-1.97, *P* < 0.001), cognitive (OR 1.27, 95% CI 1.01-1.61, *P* = 0.042), and emotional job demands (OR 1.27, 95% CI 1.02-1.58, *P* = 0.036), as well as anti-HER2 therapy (OR 2.59, 95% CI 0.90-7.49, *P* = 0.074). In the multivariable model, lower educational level (OR 4.67, 95% CI 1.08-20.2, *P* = 0.040) and higher physical job demands (OR 1.43, 95% CI 1.09-1.88, *P* = 0.009) remained independent predictors. Anti-HER2 therapy demonstrated a borderline association (OR 3.45, 95% CI 0.89-13.3, *P* = 0.074) (Table 4).

**Table 4:** Multivariable logistic regression analysis of predictors of post-treatment sick leave and prolonged sick leave (>12 months). Multivariable logistic regression adjusted for all variables shown. Variables with *P* < 0.05 were considered statistically significant. OR, odds ratio; CI, confidence interval

Variable	OR	95% CI	*P*-value
Post-treatment sick leave			
Education level ≤9th grade	4.67	1.08-20.2	0.04
Physical job demands	1.43	1.09-1.88	0.009
Anti-HER2 therapy	3.45	0.91-13.1	0.074
Cognitive job demands	1.26	0.75-2.11	0.371
Emotional job demands	1.04	0.63-1.72	0.871
Permanent contract	0.67	0.18-2.44	0.561
Prolonged sick leave (>12 months)			
Physical job demands	1.24	0.98-1.57	0.076
Education level ≤9th grade	2.43	0.76-7.76	0.137
Adjuvant radiotherapy	0.48	0.17-1.39	0.213
Cognitive job demands	1.19	0.90-1.57	0.256
Permanent contract	0.52	0.13-2.05	0.521

For prolonged sick leave (> 12 months), univariable analyses identified lower educational level (OR 2.56, 95% CI 0.94-6.97, *P* = 0.060), absence of a permanent contract (OR 0.32, 95% CI 0.12-0.86, *P* = 0.031), higher physical (OR 1.37, 95% CI 1.11-1.70, *P* = 0.004) and cognitive job demands (OR 1.23, 95% CI 0.97-1.54, *P* = 0.084), and adjuvant radiotherapy (OR 0.36, 95% CI 0.13-1.00, *P* = 0.056) as potential predictors. In the multivariable analysis (Table 4), no variable reached statistical significance, although higher physical job demands (OR 1.24, 95% CI 0.98-1.57, *P* = 0.076) demonstrated a borderline association.

Quality of life and sick leave

Women who remained on sick leave after completing treatment reported significantly higher levels of breast symptoms (mean 1.97 ± 0.90 vs. 1.38 ± 0.58; *U* = 833.0, *P* = 0.0076) and arm symptoms (mean 2.16 ± 0.92 vs. 1.67 ± 0.82; *U* = 812.5, *P* = 0.0262) compared with those who had returned to work. No statistically significant differences were observed in body image, sexual satisfaction, or future health concerns (all *P* > 0.05).

Participants experiencing prolonged sick leave (>12 months) also reported significantly higher breast symptoms (mean 2.06 ± 0.91 vs. 1.75 ± 0.85; *U* = 799.0, *P* = 0.0487), arm symptoms (mean 2.20 ± 0.86 vs. 1.75 ± 0.85; *U* = 799.0, *P* = 0.0487), and worse body image (mean 2.17 ± 1.10 vs. 1.59 ± 0.80; *U* = 813.5, *P* = 0.0298). A trend toward greater concern about future health was also observed (*U* = 775.5, *P* = 0.0758). No differences were found in sexual satisfaction between groups (*U* = 608.5, *P* = 0.813).

## Discussion

This study evaluated RTW outcomes and factors associated with sick leave among working-age women treated with chemotherapy for early-stage breast cancer. While most participants eventually returned to work, a substantial proportion experienced prolonged sick leave and reported significant physical, cognitive, and emotional challenges related to reintegration. Given the exploratory, cross-sectional design, these findings should be interpreted cautiously, particularly regarding temporal relationships and the directionality of associations.

Our cohort had an RTW rate of 81.4%, with a median time to return of eighteen months post-diagnosis. These figures situate our results within the broad international spectrum: systematic reviews have reported RTW rates ranging from 43% to 93% one year after diagnosis [6], a variability largely attributed to differences in healthcare systems, social protection policies, treatments, and patient characteristics. For instance, Peugniez et al. [15] reported an RTW rate of 79.8% with a median delay of 11.5 months over a 36-month follow-up. Although our RTW rate is comparable, the time to return appeared notably longer in our cohort. This difference may partly reflect contextual factors specific to the Portuguese labor and social security system, and the limited generalizability of our single-center sample.

Our findings also suggest long-lasting functional limitations after chemotherapy. More than seventy percent of those on sick leave remained absent after completing chemotherapy, and nearly nineteen percent had not returned to work by the time of data collection. These observations are consistent with prospective cohorts such as CANTO [16], where twenty-one percent of women had not returned two years after diagnosis, and with randomized data from the PhysSURG-B trial [17], which also reported greater sick-leave days among women receiving chemotherapy. However, potential selection bias-such as greater participation among survivors more willing or able to discuss employment, may have influenced these estimates.

Importantly, many participants did not return to work under the same conditions as before diagnosis. Several transitioned from full-time to part-time employment, changed job roles, or experienced income loss. Similar patterns were described in the CANTO cohort [16], where one in four women who initially returned later experienced interruptions, and in a systematic review by Mehnert [18], which found that 30%-40% of survivors faced adverse employment changes. In Denmark, Jensen et al. [19] similarly reported reduced income growth among survivors, particularly in the first three years after diagnosis.

The most frequently reported reasons for extended sick leave in our cohort are fatigue, pain, limited arm mobility, and neuropathy-mirror findings from large survivorship cohorts [18,20], where fatigue consistently emerges as a major factor associated with delayed RTW. Cognitive difficulties such as memory impairment, reduced concentration, and decreased task efficiency were also commonly reported, aligning with observations from the French CANTO study [16] and from Koppelmans et al. [21], underscoring the relevance of cancer-related cognitive challenges in occupational reintegration. Emotional symptoms-including mood fluctuations, diminished motivation, and poor self-image-have also been associated with prolonged absenteeism in prior reviews [9,18]. Employer-related barriers, such as inadequate workplace adjustments or insufficient flexibility, have been documented across European settings [22,23], highlighting the need for structured employer engagement. Our finding that many women viewed RTW as a way to regain identity and purpose aligns with qualitative literature emphasizing the symbolic and psychosocial value of employment [24,25].

In our cohort, lower educational level and higher physical job demands were associated with sick leave after treatment. This pattern is consistent with prior reviews [18] and registry-based studies [9] identifying socioeconomic vulnerability and physically demanding work as key correlates of work absence. For prolonged sick leave (>12 months), no factor remained significant in multivariable analysis, although higher physical job demands showed a borderline association. These trends resemble prior findings [18,25] linking physically strenuous work with delayed or less sustainable RTW. Interestingly, having a permanent contract appeared protective against prolonged absence, contrasting with its association with sick leave during treatment. This may reflect different temporal dynamics, whereby job security facilitates leave during the acute phase but supports reintegration in the longer term by providing stability and opportunities for graded return. Nonetheless, the modest sample size limits statistical power for multivariable modelling, particularly for the prolonged sick leave outcome, and small effect sizes may therefore have gone undetected.

Participants also identified physical exercise, psychological support, and postoperative physical therapy as important facilitators of RTW. Similar findings from the CANTO cohort [16] showed that access to rehabilitation and supportive care was associated with improved work outcomes. Exercise and physiotherapy have been shown to reduce fatigue and improve physical functioning [20,26], while psychological support may mitigate distress and enhance motivation [18]. These results reinforce the relevance of integrating early rehabilitation and multidisciplinary supportive interventions into survivorship pathways to promote sustainable RTW. However, as these data were self-reported, recall and perception biases must be considered when interpreting the relative importance of these facilitators.

Women on sick leave reported lower quality of life, largely due to greater breast and arm symptom burden. Among those with prolonged sick leave, worse breast and arm symptoms and poorer body image were observed, along with a trend toward greater concern about future health. Similar findings in the CANTO cohort [16] identified breast and arm morbidity as factors associated with long-term work disruption, and other studies [27,28] have highlighted body-image and sexual concerns as relevant barriers to RTW.

In summary, this study illustrates the multifactorial nature of work reintegration after breast cancer. RTW appears to be a dynamic process shaped by clinical, psychosocial, and occupational factors. Given the exploratory and cross-sectional design, the associations identified should not be interpreted as determinants. Instead, they generate hypotheses that warrant confirmation in longitudinal and multicenter studies. Individualized and multidisciplinary survivorship care is essential to support functional recovery and promote sustainable participation in the workforce.

Strengths and limitations

This study provides novel insights into work outcomes after breast cancer in a Portuguese population, representing one of the first systematic mappings of a regional cohort. By combining clinical, psychosocial, and occupational variables, it offers valuable real-world evidence to inform survivorship care and workplace reintegration strategies.

Some limitations should be acknowledged. This analysis reflects data from a single center within the multicenter RESTART project, which explains the smaller sample size compared with the initial calculation of 384 participants. The modest sample size and the relatively small number of events, particularly for prolonged sick leave, may have reduced statistical power and limited the detection of weaker associations. Although the reduced cohort (*n* = 71) may limit statistical power to detect weaker associations, it still provides a meaningful snapshot of a representative regional population. The cross-sectional design also precludes causal inference, and several variables were self-reported, which may introduce recall bias. Potential selection bias and healthy worker effects may also influence the observed estimates, and generalizability beyond the regional Portuguese context is limited. Despite these considerations, this study contributes important data in an underexplored context and highlights the need for future multicenter and longitudinal studies.

## Conclusions

Breast cancer survivors face multiple challenges in returning to work, even after completing active treatment. Although most women in this study eventually resumed employment, many reported persistent physical, cognitive, and emotional symptoms that limited their ability to return fully or maintain their previous work conditions. In the multivariable analysis, lower educational level and higher physical job demands independently predicted sick leave after treatment. For prolonged sick leave (>12 months), no variable reached statistical significance, although higher physical job demands showed a borderline association. Survivors also frequently perceived limited workplace support as a barrier to reintegration.

These findings highlight the need for multidisciplinary survivorship care that integrates RTW planning from an early stage. Identifying individuals at increased risk of work disruption and fostering coordinated communication between healthcare providers, patients, and employers may help promote sustainable work participation and improve quality of life for breast cancer survivors.

Given the single-center, cross-sectional design and modest sample size, the results should be interpreted as exploratory and hypothesis-generating. Future multicenter longitudinal studies are needed to validate these associations and better characterize long-term work reintegration trajectories after breast cancer.
